# Post-traumatic Exacerbation of Grade II L5-S1 Spondylolisthesis Requiring Surgical Stabilization

**DOI:** 10.7759/cureus.108446

**Published:** 2026-05-07

**Authors:** Elizabeth Blanco Espinosa, Idania Cruzata Matos, Yelka Matos Furones, Ana B Cuni Hernandez, Ricardo Marlon Saro Del Valle, Abel Diaz

**Affiliations:** 1 Neurosurgery/General Surgery, Arnaldo Milián Castro Provincial University Hospital, Santa Clara, CUB; 2 General Practice, Ceda Orthopedic Group, Miami, USA; 3 General Medicine, HCA Healthcare, Nevada, USA; 4 Neurology, North Georgia Clinical Research/Alcanza Clinical Research, Woodstock, USA; 5 Family Medicine, Atlas Urology, Bradenton, USA; 6 Neurology, Dr. Juan Bruno Zayas Alfonso General Hospital, Santiago de Cuba, CUB; 7 Cardiology, Universidad de Ciencias Médicas de Matanzas, Matanzas, CUB

**Keywords:** case report, l5–s1, low-energy trauma, lumbar spine, paresthesia, pedicle screw fixation, posterior lumbar fusion, spinal instability, spinal instrumentation, spondylolisthesis

## Abstract

Spondylolisthesis is characterized by the forward movement of one vertebra over the one beneath it, most frequently occurring at the L5-S1 level due to its biomechanical susceptibility. Although often asymptomatic, it may become clinically significant when underlying instability is exacerbated by external factors. Low-energy trauma can unmask previously undiagnosed spondylolisthesis, resulting in acute pain, neurological symptoms, and functional impairment. We report the case of a 51-year-old woman with a history of hypertension treated with atenolol, with no prior medically documented history of chronic low back pain or spinal pathology (noting that this information was obtained retrospectively from patient history after resolution of acute intoxication). She presented with severe low back pain following a fall from standing height while under the influence of alcohol. The pain was progressive, refractory to analgesia, with a reported intensity of 9/10 on the Visual Analog Scale (VAS), and associated with paresthesia and impaired ambulation. No standardized functional outcome scores were available pre- or post-operatively, which limits functional outcome comparison. Imaging studies, including magnetic resonance imaging (MRI), revealed Grade II L5-S1 spondylolisthesis with degenerative disc changes, canal narrowing, and suspected neural compression, without evidence of acute fracture. Computed tomography (CT) was not available at presentation for independent review, and prior external imaging reports were not retrievable.

Given persistent symptoms after failed conservative management and clinical evidence of neurological progression, the patient underwent posterior lumbar stabilization with pedicle screw instrumentation at L5-S1. The procedure performed was posterior instrumented fusion with pedicle screw fixation and posterolateral arthrodesis. The postoperative course was uneventful, with improvement in pain and mobility and resolution of sensory symptoms. The VAS score improved from 9/10 preoperatively to 2/10 at the six-week follow-up. The patient was discharged on postoperative day 3 and showed continued improvement at six months.

This case highlights that low-energy trauma may reveal previously asymptomatic spondylolisthesis. Early recognition, appropriate imaging, and timely surgical intervention are essential for optimal outcomes. Posterior instrumentation remains a reliable treatment option when tailored to clinical presentation. However, functional outcome measures and long-term radiographic fusion assessment were not available, limiting interpretation of definitive recovery.

## Introduction

Spondylolisthesis is defined as the anterior displacement of a vertebral body relative to the one below it, most commonly occurring at the lumbosacral junction, particularly at the L5-S1 level, driven by the unique biomechanical stresses and orientation of spinopelvic anatomy at this segment [1]. It represents a relatively common spinal condition that may remain asymptomatic; epidemiological studies suggest that slippage and associated pars lysis vary widely within populations and often go undiagnosed until symptomatic [2].

The etiology of spondylolisthesis is multifactorial and includes degenerative changes in the intervertebral discs and facet joints, defects of the pars interarticularis (spondylolysis), congenital anomalies, dysplastic abnormalities, and, less commonly, acute traumatic causes [1].

Spinopelvic alignment parameters such as pelvic incidence, pelvic tilt, and sacral slope have been described in the literature as contributors to sagittal balance and may influence mechanical stress distribution at the lumbosacral junction [3]. These parameters contribute to the biomechanical environment that predisposes individuals to vertebral slippage by altering sagittal balance and shear forces at L5-S1 [4]. However, they are primarily relevant in preoperative planning and chronic deformity assessment and are not routinely available in acute trauma settings.

Several risk factors contribute to the development and progression of spondylolisthesis, including age-related degeneration, female sex, repetitive lumbar extension activities, obesity, and spinopelvic alignment abnormalities [1]. In many patients, the condition remains asymptomatic for prolonged periods. However, external triggers such as low-energy trauma may precipitate acute symptom onset by unmasking underlying instability [5].

Low-energy trauma can therefore reveal previously asymptomatic or undiagnosed spondylolisthesis, resulting in acute pain, instability, and neurological symptoms [6]. Imaging plays a central role in evaluation. Computed tomography (CT) is considered the most accurate modality for assessing bony alignment, pars defects, and degree of slippage, while MRI is essential for evaluating neural compression and disc degeneration [2]. In this case, magnetic resonance imaging (MRI) was the primary imaging modality available for clinical decision-making, as prior CT studies were not accessible for review at the time of admission.

Management depends on symptom severity and the presence of instability or neurological compromise. While conservative treatment may be appropriate in selected cases, surgical intervention is indicated in patients with persistent pain, functional limitation, or neurological deficits [1].

In this report, we present a case of unstable L5-S1 spondylolisthesis unmasked by low-energy trauma and managed with posterior spinal instrumentation following clinical deterioration. Despite its relatively high prevalence, there is limited literature describing the acute symptomatic presentation of previously undiagnosed spondylolisthesis triggered by low-energy trauma.

## Case presentation

History and clinical findings

A 51-year-old woman with a history of hypertension, treated with atenolol, presented to the emergency department approximately two months after sustaining a fall from standing height while under the influence of alcohol. Following the initial injury, the patient was evaluated at an outside facility where a CT scan and X-ray imaging were reportedly performed, and she was discharged with conservative management including analgesics. However, the original imaging reports and detailed radiological findings were not available for review at the time of this admission. The clinical history, including absence of prior low back pain or known spinal pathology, was obtained after resolution of the acute intoxication state, improving the reliability of patient reporting.

Over the following weeks, the patient experienced persistent and progressively worsening low back pain. At two months post-injury, she re-presented with severe refractory pain, paresthesias in the lower extremities, and new-onset urinary incontinence.

On examination, the patient was hemodynamically stable. There was marked tenderness over the lumbosacral region with restricted range of motion due to pain. Neurological examination revealed preserved motor strength (5/5) in all major muscle groups of the lower extremities. Sensory examination demonstrated decreased sensation to light touch and pinprick in the L5-S1 dermatomal distribution. The straight leg raise test was positive at 45 degrees. Deep tendon reflexes were normal. The patient also reported new-onset urinary incontinence, raising concern for evolving neurological compromise. Gait was limited due to pain and sensory symptoms.

Imaging findings

**Figure 1:**
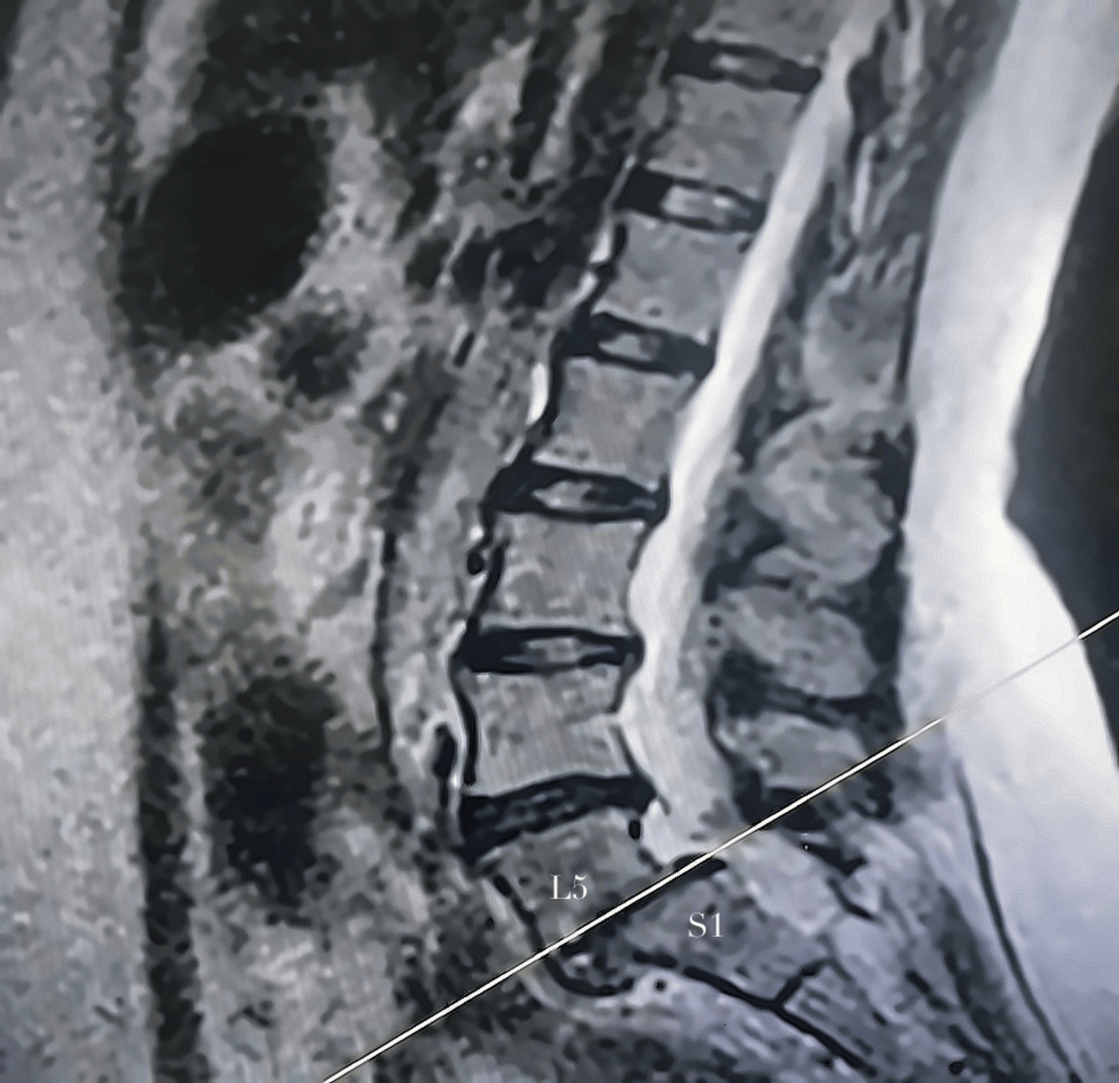
Lumbar spine MRI, sagittal view, demonstrating anterior displacement of L5 over S1 consistent with spondylolisthesis (white line)

MRI of the lumbar spine demonstrated anterior displacement of the L5 vertebral body over S1, consistent with Grade II spondylolisthesis (Meyerding classification), with approximately 35% slippage. No acute fracture was identified. MRI also revealed degenerative disc disease at L5-S1 with canal narrowing and suspected neural element compression. Dynamic flexion-extension radiographs were not obtained due to pain-limited mobility (Figure 1). 

Although spinopelvic parameters such as pelvic incidence, pelvic tilt, and sacral slope are relevant in the comprehensive assessment of spondylolisthesis, these measurements were not obtained due to the absence of dedicated preoperative full-spine lateral imaging. Therefore, surgical decision-making was based on clinical presentation, neurological status, and segmental imaging findings rather than global spinopelvic alignment.

Management and surgical indication

The patient had undergone approximately two months of conservative management after the initial injury, including analgesics and activity modification, without clinical improvement. Upon re-presentation, she had severe refractory low back pain, functional limitation with impaired ambulation, persistent sensory symptoms, new-onset urinary incontinence, and imaging findings consistent with degenerative spondylolisthesis and neural compression. Given the failure of prolonged conservative treatment and clinical deterioration, surgical management was indicated.

The patient was admitted and managed with continued conservative measures; however, due to persistent symptoms and neurological progression, surgery was performed approximately 15 days after admission following multidisciplinary evaluation.

Surgical technique

**Figure 2:**
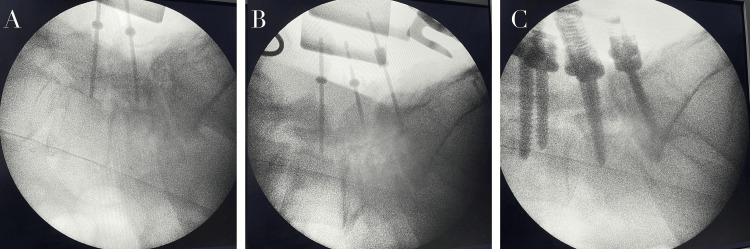
Intraoperative fluoroscopic guidance during posterior lumbar instrumentation (A) Initial pedicle cannulation under fluoroscopy, (B) advancement of pedicle screws confirming appropriate trajectory, (C) final positioning of pedicle screws and rod construct, demonstrating adequate alignment and stabilization.

The patient was positioned prone on a radiolucent table. A standard midline posterior approach was used to expose the posterior elements of L5 and S1. Pedicle entry points were identified using anatomical landmarks at the junction of the superior articular facet and transverse process. Pedicles were cannulated using a probe with approximately 15-20° medial angulation, maintaining a trajectory parallel to the superior endplate. After cannulation, the integrity of the pedicle walls and the tract base was carefully assessed with a ball-tipped probe to confirm the absence of cortical breach before screw placement. Pedicle screws were then inserted bilaterally at L5 and S1 under fluoroscopic guidance (Figure 2).

**Figure 3:**
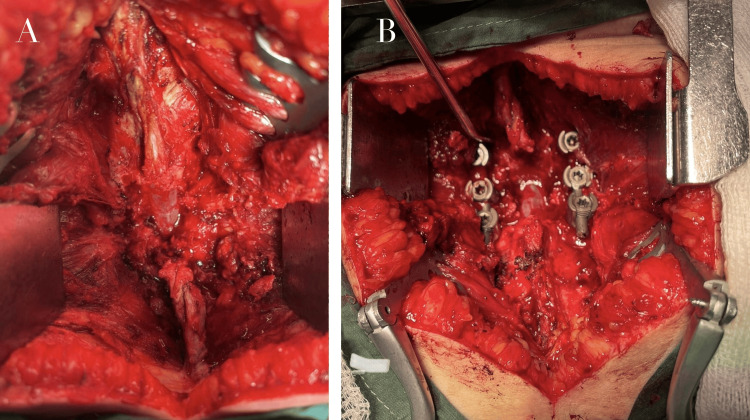
Intraoperative posterior lumbar instrumentation (A) Exposure of posterior elements at L5-S1 via a midline posterior approach, (B) bilateral pedicle screw placement and rod fixation under fluoroscopic guidance, achieving stabilization of the spondylolisthesis.

Posterior instrumented fusion (L5-S1) with pedicle screw fixation and posterolateral arthrodesis was performed. Rods were subsequently placed and secured, achieving stabilization and partial reduction of the spondylolisthesis. The wound was irrigated and closed in layers. No intraoperative complications were observed (Figure 3).

**Figure 4:**
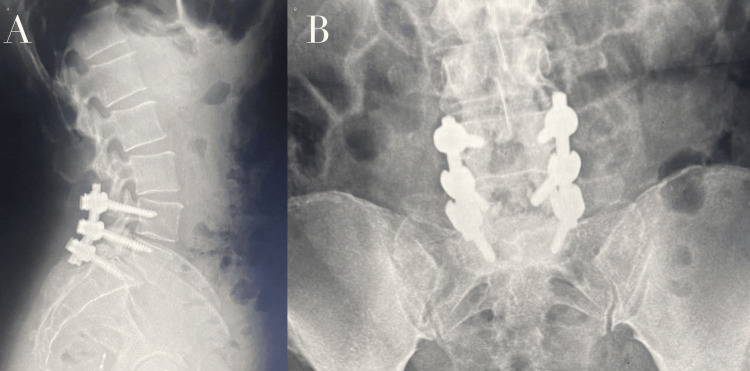
Postoperative imaging after instrumented posterior fusion at L5-S1 (A) Lateral X-ray showing the final construct with pedicle screw fixation and restoration of alignment. (B) Anteroposterior pelvic radiograph confirming appropriate positioning of the fixation hardware at L5-S1.

Postoperatively, the patient showed significant improvement in pain and mobility, with resolution of paresthesias. Neurological status remained stable, and urinary symptoms improved clinically. The patient was discharged on postoperative day 3. There was improvement in pain, with a reduction in the Visual Analog Scale (VAS) score from 9/10 preoperatively to 2/10 at follow-up. Follow-up was extended to six months, during which the patient maintained clinical improvement and functional recovery. Radiographs obtained at two months postoperatively demonstrated stable hardware positioning and maintained spinal alignment (Figure 4).

However, the interpretation of long-term outcomes is limited by the absence of validated functional outcome measures (such as the Oswestry Disability Index (ODI) or 36-Item Short Form Survey (SF-36)) and the lack of long-term radiographic assessment beyond six months to fully evaluate fusion status. In addition, initial external imaging reports were not available for complete review, which limits comprehensive correlation with the initial injury evaluation.

## Discussion

This case illustrates how an otherwise clinically silent degenerative spondylolisthesis may become symptomatic following low-energy trauma, revealing underlying segmental instability. Although spondylolisthesis is commonly associated with chronic low back pain, acute presentations may occur when underlying instability is exacerbated by external factors [7]. Alcohol intoxication is a well-recognized risk factor for falls and may contribute to both the mechanism of injury and delayed presentation. Clinicians should maintain a high index of suspicion for spinal instability in such patients, even in apparently minor trauma scenarios [8].

The L5-S1 segment is biomechanically vulnerable due to its transitional role and exposure to high shear forces [9]. In this patient, the absence of an acute fracture supports the presence of a pre-existing degenerative instability that became clinically symptomatic following trauma.

From a diagnostic standpoint, CT and MRI are complementary modalities in the evaluation of spondylolisthesis. CT provides superior assessment of osseous alignment and vertebral translation, while MRI is essential for evaluating neural compression and disc degeneration [10]. However, in this case, prior CT imaging reports from the initial external evaluation were not available for complete review, and management was guided primarily by MRI findings and clinical status.

Posterior instrumented fusion with pedicle screw fixation and posterolateral arthrodesis remains a well-established technique, providing immediate stability and allowing partial reduction of the slip. In this case, surgical intervention was indicated due to progressive neurological deterioration, including urinary incontinence, persistent severe pain, functional impairment, and failure of initial conservative management, rather than elective or purely pain-driven management [7]. Although interbody fusion techniques such as transforaminal lumbar interbody fusion (TLIF), posterior lumbar interbody fusion (PLIF), and anterior lumbar interbody fusion (ALIF) are widely used for restoration of disc height and sagittal alignment, outcomes compared to posterior-only approaches are often similar in appropriately selected patients [11,12]. Minimally invasive techniques such as minimally invasive surgery for TLIF (MIS-TLIF) have demonstrated reduced blood loss and shorter hospital stay while maintaining comparable fusion rates [13]. However, these approaches require specific expertise and are not universally indicated. In this case, posterior-only fixation with posterolateral fusion was selected as it provided sufficient stabilization in the setting of neurological progression and allowed timely decompression and stabilization without anterior column reconstruction.

Spinopelvic parameters (pelvic incidence, pelvic tilt, sacral slope) were not available due to the lack of dedicated full-spine imaging and therefore could not be incorporated into preoperative planning. This represents a methodological limitation and is now explicitly acknowledged as it prevents full biomechanical characterization of sagittal alignment. However, their absence did not alter the urgent clinical decision-making process, which was driven by neurological deterioration. An additional relevant aspect is the absence of prior symptoms despite Grade II spondylolisthesis, suggesting that significant vertebral slippage may remain clinically silent until a triggering event occurs.

A major limitation of this study is the reliance on VAS as the primary quantitative outcome measure. Validated functional disability scores such as ODI, SF-36, or PROMIS (Patient-Reported Outcomes Measurement Information System) were not available, which limits objective assessment of functional recovery and comparison with the existing literature. Other limitations include its single-patient design and limited follow-up duration. Although the patient demonstrated significant clinical improvement at six months, long-term outcomes such as definitive fusion status, pseudarthrosis, or adjacent segment disease cannot be fully assessed.

## Conclusions

Low-energy trauma may be associated with previously asymptomatic or undiagnosed spondylolisthesis, resulting in acute pain, neurological symptoms, and functional impairment. This case emphasizes the importance of maintaining a high index of suspicion for underlying spinal instability, even in apparently minor trauma scenarios. Early and comprehensive evaluation with appropriate imaging modalities, including CT and MRI, is essential for accurate assessment of structural and neural involvement.

In patients with persistent pain, neurological symptoms, or radiographic evidence of instability, timely surgical intervention may be considered to achieve stabilization and symptom relief. In this case, posterior instrumented fusion with pedicle screw fixation and posterolateral arthrodesis was performed based on clinical deterioration, failure of initial conservative management, and progression of neurological symptoms, resulting in significant short-term clinical improvement. However, the absence of standardized functional outcome measures and the limited follow-up period (six months) prevented definitive assessment of long-term functional recovery and fusion status. This highlights the inherent limitations of single-case reports in evaluating the durability of surgical outcomes. Overall, an individualized approach integrating clinical presentation, imaging findings, and patient-specific factors remains essential for the optimal management of spondylolisthesis in an acute setting.
